# LoRTE: Detecting transposon-induced genomic variants using low coverage PacBio long read sequences

**DOI:** 10.1186/s13100-017-0088-x

**Published:** 2017-04-08

**Authors:** Eric Disdero, Jonathan Filée

**Affiliations:** Laboratoire Evolution, Génomes, Comportement, Ecologie; CNRS, IRD, Université Paris-Saclay, Gif-sur-Yvette, France

**Keywords:** Transposable element, Structural variation, Population genomic, Long read sequence

## Abstract

**Background:**

Population genomic analysis of transposable elements has greatly benefited from recent advances of sequencing technologies. However, the short size of the reads and the propensity of transposable elements to nest in highly repeated regions of genomes limits the efficiency of bioinformatic tools when Illumina or 454 technologies are used. Fortunately, long read sequencing technologies generating read length that may span the entire length of full transposons are now available. However, existing TE population genomic softwares were not designed to handle long reads and the development of new dedicated tools is needed.

**Results:**

LoRTE is the first tool able to use PacBio long read sequences to identify transposon deletions and insertions between a reference genome and genomes of different strains or populations. Tested against simulated and genuine *Drosophila melanogaster* PacBio datasets, LoRTE appears to be a reliable and broadly applicable tool to study the dynamic and evolutionary impact of transposable elements using low coverage, long read sequences.

**Conclusions:**

LoRTE is an efficient and accurate tool to identify structural genomic variants caused by TE insertion or deletion. LoRTE is available for download at http://www.egce.cnrs-gif.fr/?p=6422

## Background

Transposable elements (TEs), which represent an essential part of eukaryotic and prokaryotic genomes, play important roles in genome size, structure and functions [1, 2]. TE identification and annotation remains one of the most challenging task in computational genomics [3, 4] but our knowledge of the TE diversity and dynamics among genomes has greatly benefited from the recent advance of sequencing technologies [3]. Specifically, comparison of closely related strains or species using short read sequencing technologies enabled new insights into TE dynamic and their roles in generating structural genomic variation. Two different approaches with their associated computational tools have been developed to achieve this goal, see [5, 6] for exhaustive descriptions of the different strategies. Briefly, the first approach is based on the direct assembly of the repeated fraction of the reads using highly abundant k-mer : RepARK [4] or Tedna [7]. Other tools such as RepeatExplorer [8] or dnaPipeTE [9] used low-coverage sub-samples of the reads in order to retrieve and specifically assemble the highly repeated elements. All these tools have the advantage to give a good picture of the global TE abundance and diversity. However they do not provide the exact genomic positions of each TE, preventing the identification of the presence/absence of given TE copies between related populations or species. The second approach is implemented in programs that have been specifically developed to detect transposon presence/absence between a reference genome and Illumina or 454 short read sequences [10–13]. The global architecture of these softwares is similar: 1. New insertions are detected by retrieving the reads that do not map on the reference genomes but that align both on a TE consensus sequence and a unique region in the genome. 2. Deletions are detected by identifying reads that align on the two flanking sequences of a given TE present in the reference genome indicating that the locus not contains anymore the sequence of the TE copy. Programs like the Transposon Insertion and Depletion AnaLyzer (TIDAL) also take advantage of the presence of paired end sequences on Illumina reads to identify the deleted locus [12]. This later approach has been extensively tested and benchmarked on diverse *Drosophila* datasets leading to mixed results. Indeed, comparison of respective performance of each program indicated that a very small fraction of the TE presence/absence was identified by all programs [12, 13]. For example, the comparison of TIDAL [12], TEMP [13], LnB [14] and CnT [15] on Drosophila Synthetic Population Resource (DGRP) strains [16] revealed that only 3% of the calls are predicted in common by the different programs. Thus, a large majority of the predictions are program-specific and PCR validations of the calls lead to substantial levels of false positive (around 40%) [12]. These limitations are mainly due to the fact that TEs tend to insert preferentially in highly repetitive regions. The short length of Illumina reads prevents the precise identification and mapping of these TEs nested in one another. Additionally, the precise breakpoint prediction required the use of specific softwares [17]. Interestingly, long read sequencing technologies such as those provided by PacBio or MinION technologies are now generating read length that may span the entire length of full transposons and their associated flanking genomic sequences. However, existing programs are not designed to deal with long read sequences and the implementation of new methods is thus required. Here we present LoRTE (Long Read Transposable Element), the first tool for population genomic analyses of TE presence/absence between a reference genome and PacBio long read sequences.

## Implementation

**Fig. 1:**
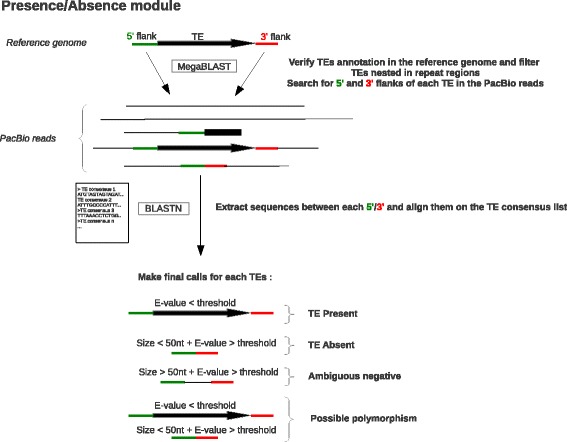
Simplified workflow of the Presence/Absence module. *Green* and *red bars* indicate different flanking sequences, large *black arrows* represent TEs

LoRTE is a Python 2.7 program composed of two main modules (Fig. 1) that only required BLAST+ suite and BioPython as dependencies:

1. The first module is designed to verify the presence/absence in the PacBio reads of a list of annotated TEs in the reference genome (Fig. 1a). Briefly, the program acquires the flanking sequences of each TEs and align them on the reference genomes using MEGABLAST [18] (not shown in Fig. 1a). The length of the flanking sequences is specified by the user (default = 200 bp). At this stage, a filter verifies if the TE is correctly annotated and if the flanking sequences map uniquely on the genome. TE wrongly annotated or located in region too much enriched in repeats are categorized as “irresolvable locus” in the final output file. The remaining 3′ and 5′ flanking sequences are aligned on the PacBio read using MEGABLAST (Fig. 1a). All the sequences located between a 3′ and 5′ flanking sequences in the same orientation, and in a specified window size in the PacBio reads are extracted. These extracted sequences are then searched with BLASTN against the TE consensus sequences. For a given locus if the sequence matches to the same TE consensi, the TE is considered as “TE Present” in the read. Sequences <50 nt without any match on the TE consensi correspond to a deletion (“TE absent”). “Possible polymorphism” locus corresponds to a situation in which a given TE is “absent” in some reads and “present” in some others (heterozygosity or true polymorphism if the DNA of several organisms have been pooled and sequenced). Finally some locus are characterized as “ambiguous negative” if the extracted sequences between the 3′ and the 5′ flanking are >50 nt but do not match with a TE consensus sequences. This latter case may correspond to partially deleted TEs.
2. The second step aims to identify new TE insertions present in the reads but absent in the reference genome. The program removes from the PacBio reads the segments of sequences corresponding to the TEs identified by the first module. Then, the TE consensi are aligned using BLASTN on the reads to identify all the remaining TEs. The flanking 5′ and 3′ ends of these putative new TE insertions are extracted and aligned using MEGABLAST on the reference genome. All the sequences between a 5′ and 3′ ends, in the same orientation, and in a specified window size are extracted and the program verifies if they match with a TE consensus using BLASTN. If the extracted sequences are <50 nt and do not resemble to a given consensus the program considers these cases as new insertions in the reads. “New polymorphic TE insertion” corresponds to a situation in which a new previously identified TE insertion in step 1 is “present” in some reads but “absent” in some others. Finally, all the reads testifying for a new insertion for the same locus are clustered together.

To assess the performance and accuracy, we have tested LoRTE on two *Drosophila melanogaster* datasets: (i) Benchmark of the program is monitored by random insertion of 250 TEs and random deletion of 100 TEs in the reference genome (release 5) before its segmentation in pieces of 3 to 30 kb in length. More realistic, error-prone, PacBio reads have also been generated using the PBSIM software with default parameters except –length-min = 1000 [19] (ii) genuine PacBio reads of pooled 1950 adult males of the ISO1 strains (same stock used in the official reference assembly) [20] with a sequencing depth of 90× (average read length: 10,040 bp).

In order to identify false positives, LoRTE predictions are then compared with the genome assembly of the PacBio reads. Reads and the Falcon assembly [21] are available at https://github.com/PacificBiosciences/DevNet/wiki/Drosophila-sequence-and-assembly. To test the impact of the coverage on the performance of LoRTE we have sub-sampled the datasets to lower coverages (from 1× to 40×). For these experiments, we have used a list of 4239 annotated TEs [22] and corresponding TE consensi obtained from FlyBase FB2016_04 release (http://flybase.org/) and RepBase version 31/01/2014 (http://www.girinst.org/repbase/). Input and raw output files used in this study are available at http://www.egce.cnrs-gif.fr/?p=6422

LoRTE predictions on the ISO1 PacBio reads have been evaluated using the *de novo* 90× Falcon assembly. For the new TE insertions and deletions, each 3′ and 5′ flanking sequences of the corresponding predictions in the PacBio reads are aligned on the Falcon assembly using MEGABLAST. The sequences located between these 3′ and 5′ flanking sequences are extracted and searched with BLASTN against the TE consensus sequences. BLAST output files are then manually compared with the LoRTE calls to estimate the validity of each prediction.

## Results

**Fig. 2:**
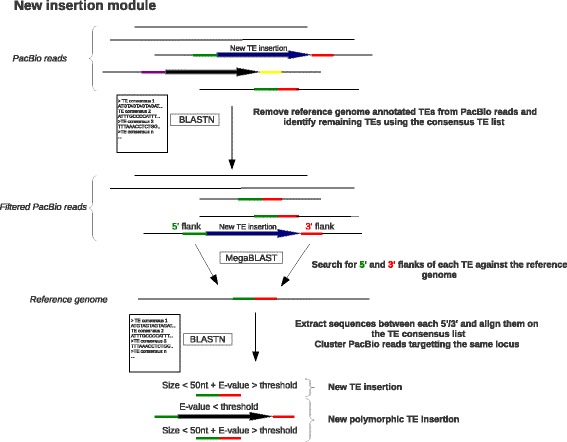
Simplified workflow of the New insertion module. *Green*, *red*, *yellow* and *purple bars* indicate different flanking sequences, *large black* and *blue arrows* represent TEs

As existing softwares designed to detect TE-induced genomic variations are not able to handle long read sequences, it is virtually impossible to compare the respective performances of LoRTE with these tools. However, LoRTE was carefully benchmarked on two different *D. melanogaster* PacBio datasets. The first is a synthetic dataset composed of 3 to 30 kb PacBio-like reads generated from the reference genome in which we inserted and deleted respectively 100 and 250 TEs. The second is a real biological dataset with *D. melanogaster* PacBio reads coming from pooled individuals of the same strain used in the reference genome. We first tested the ability of LoRTE to provide variant calls on a list of 4239 annotated TEs with respect to the read coverage (Fig. 2a). For both datasets, LoRTE was able to provide a decision for >99% of the TE locus with a coverage of 9×. Due to the relatively high error rate of the genuine PacBio raw read (around 10%, mainly short insertion/deletion events) leading to MEGABLAST misalignments, synthetic reads performed better at low coverage. Moreover, LoRTE achieved a complete analysis of the data with 10× coverage on a standard computer with 2 cores running at 2.3 GHz in less than 48 h, using a maximum of 8 Gb of RAM. This result indicate that a low PacBio read coverage, corresponding to a single single-molecule real-time (SMRT) cell generating 500 to 1000 Mb of sequences, is sufficient to make a call on the vast majority of the TE identified in the *D. melanogaster* genome.

We then tested the ability of LoRTE to detect the insertions/deletions made on the synthetic datasets. Figure 2b displays the percentage of insertions/deletions detected by LoRTE with respect to the read coverage. LoRTE detected 98% of the deletions and 100% of the insertion from coverage of 9× and did not generated false positive calls, whatever the coverage. We have also tested LoRTE with the synthetic datasets generated by the PBSIM software [19] that simulates the size distribution and the high error rate of genuine PacBio reads. With a coverage of 10×, we obtained very similar results using error-free and PBSIM error-prone PacBio reads. The detection of the deletion appears slightly less efficient with error-prone reads, mainly because the alignments of the flanking 5′ and 3′ sequences of each TE locus generate some misalignments. This phenomenon leads to the extraction of some sequences located between these 5′ 3′ that are longer than the threshold of 50 nt. Consequently, these loci appear as «ambiguous negative >50 nt» or «possible polymorphism» rather than «TE absent». By relaxing the threshold at 100 nt, most of these loci now appear as «TE absent». However, on real PacBio reads, a relaxation of this threshold could generate false positives or an overestimation of the level of polymorphism. Taken together, these results strengthen the reliability of LoRTE, even in a context of low coverage PacBio datasets.

**Fig. 3:**
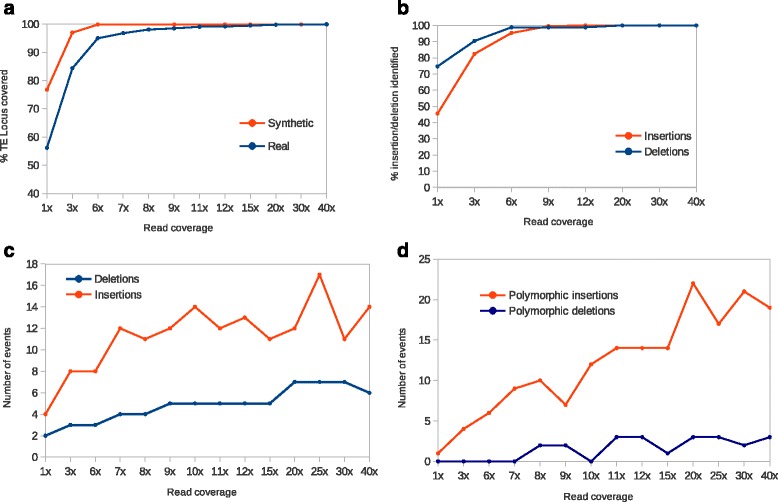
Performance test of LoRTE according to the PacBio read coverage. **a** Percentage of the TEs annotated in the *Drosophila melanogaster* genome that have been recovered by the program. **b** Percentage of the insertion/deletion artificially made in the synthetic reads that have been identified. **c** Numbers of new TE deletion and insertion found in the genuine reads and absent in the reference genome. **d** Numbers of polymorphic TE deletion and insertion found in the real PacBio reads and absent in the reference genome

**Fig. 4:**
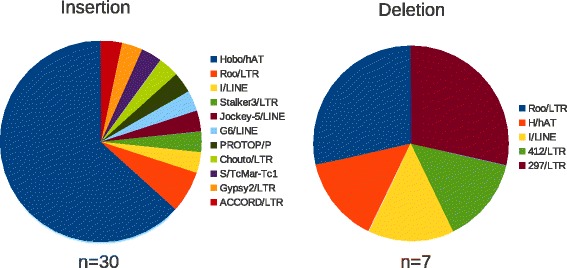
Family distribution of the total number of new TE insertion and deletion found whatever the read coverage in the *Drosophila melanogaster* PacBio reads and absent in the reference genome. Polymorphic/heterozygous events are included

We finally analyzed the results obtained by LoRTE on genuine *D. melanogaster* PacBio reads and compared the predictions with the Falcon 90× PacBio assembly. Figure 2c shows the number of deletion/insertion found in these reads. The number of deletions was relatively constant whatever the read coverage considered. With a coverage of 40×, we identifed a maximum of seven deletions corresponding mainly to LTR retrotransposons (two *roo,* two *297*, one *412*), one LINE (*I* element) and one hAT DNA transposon (Fig. 3). All of these deletions were present in the 90× genome assembly suggesting that these variants are *bona fide* TE deletions that were not present in the reference genome. Conversely, the number of new TE insertions observed in the PacBio reads increases linearly and reach a plateau from a read coverage of 10× corresponding to number of 12 to 17 new insertions (Fig. 2c). Among the 14 new insertions identified using a coverage of 40×, 12 were validated in the 90× Falcon PacBio genome assembly. The remaining 2 insertions most probably correspond to polymorphic events. Analysis of the polymorphic events (Fig. 2d) showed that the number of polymorphic insertion increase linearly with the read coverage whereas the quantity of polymorphic deletion remains at a very low level. The vast majority of these new insertions are due to *Hobo* elements, a hAT DNA transposon known to have been recently acquired in *D. melanogaster. Hobo* elements are subject to a fast and ongoing expansion in the genome and might generated frequent cut-and-paste in somatic tissues [23] (Figs. 3 and 4). Almost all of the polymophic insertions/deletions were absent in the assembly and their calls are generally supported by only one or a few PacBio reads. Thus, the calls classified as polymorphic most probably result from somatic insertions/deletions at low frequencies but possible false positives could not be ruled out.

## Conclusion

Taken together, our results indicate that LoRTE is an efficient and accurate tool to identify structural genomic variants caused by TE insertion or deletion among closely related populations or strains. Here, we demonstrated that LoRTE performs well even at low coverage PacBio read (<10×) providing a cost effective tool to study the dynamics and impact of TEs in natural populations.

## Abbreviations

TE: Transposable element

## References

[1] Fedoroff NV (2012). Presidential address. Transposable elements, epigenetics, and genome evolution. Science.

[2] Hua-Van A, Le Rouzic A, Boutin TS, Filee J, Capy P (2011). The struggle for life of the genome’s selfish architects. Biol Direct.

[3] Lerat E (2010). Identifying repeats and transposable elements in sequenced genomes: how to find your way through the dense forest of programs. Heredity (Edinb).

[4] Koch P, Platzer M, Downie BR (2014). RepARK--de novo creation of repeat libraries from whole-genome NGS reads. Nucleic Acids Res.

[5] Ewing AD (2015). Transposable element detection from whole genome sequence data. Mob DNA.

[6] Rishishwar L, Mariño-Ramírez L, Jordan IK. Benchmarking computational tools for polymorphic transposable element detection. Briefings Bioinf. 2016. bbw072. https://academic.oup.com/bib/article-abstract/doi/10.1093/bib/bbw072/2562836/Benchmarkingcomputational-tools-for-polymorphic?redirectedFrom=fulltext. 10.1093/bib/bbw072 PMC5808724 27524380

[7] Zytnicki M, Akhunov E, Quesneville H (2014). Tedna: a transposable element de novo assembler. Bioinformatics.

[8] Novak P, Neumann P, Pech J, Steinhaisl J, Macas J (2013). RepeatExplorer: a Galaxy-based web server for genome-wide characterization of eukaryotic repetitive elements from next-generation sequence reads. Bioinformatics.

[9] Goubert C, Modolo L, Vieira C, ValienteMoro C, Mavingui P, Boulesteix M (2015). De novo assembly and annotation of the Asian tiger mosquito (Aedes albopictus) repeatome with dnaPipeTE from raw genomic reads and comparative analysis with the yellow fever mosquito (Aedes aegypti). Genome Biol Evol.

[10] Fiston-Lavier AS, Barron MG, Petrov DA, Gonzalez J (2015). T-lex2: genotyping, frequency estimation and re-annotation of transposable elements using single or pooled next-generation sequencing data. Nucleic Acids Res.

[11] Kofler R, Betancourt AJ, Schlotterer C (2012). Sequencing of pooled DNA samples (Pool-Seq) uncovers complex dynamics of transposable element insertions in Drosophila melanogaster. PLoS Genet.

[12] Rahman R, Chirn GW, Kanodia A, Sytnikova YA, Brembs B, Bergman CM, Lau NC (2015). Unique transposon landscapes are pervasive across Drosophila melanogaster genomes. Nucleic Acids Res.

[13] Zhuang J, Wang J, Theurkauf W, Weng Z (2014). TEMP: a computational method for analyzing transposable element polymorphism in populations. Nucleic Acids Res.

[14] Linheiro RS, Bergman CM (2012). Whole genome resequencing reveals natural target site preferences of transposable elements in Drosophila melanogaster. PLoS One.

[15] Cridland JM, Macdonald SJ, Long AD, Thornton KR (2013). Abundance and distribution of transposable elements in two Drosophila QTL mapping resources. Mol Biol Evol.

[16] Mackay TF, Richards S, Stone EA, Barbadilla A, Ayroles JF, Zhu D, Casillas S, Han Y, Magwire MM, Cridland JM (2012). The Drosophila melanogaster genetic reference panel. Nature.

[17] Hénaff E, Zapata L, Casacuberta JM, Ossowski S (2015). Jitterbug: somatic and germline transposon insertion detection at single-nucleotide resolution. BMC Genomics.

[18] Johnson M, Zaretskaya I, Raytselis Y, Merezhuk Y, McGinnis S, Madden TL (2008). NCBI BLAST: a better web interface. Nucleic Acids Res.

[19] Ono Y, Asai K, Hamada M (2013). PBSIM: PacBio reads simulator—toward accurate genome assembly. Bioinformatics.

[20] Kim KE, Peluso P, Babayan P, Yeadon PJ, Yu C, Fisher WW, Chin C-S, Rapicavoli NA, Rank DR, Li J. Long-read, whole-genome shotgun sequence data for five model organisms. Sci Data. 2014;1.140045 10.1038/sdata.2014.45 PMC4365909 25977796

[21] Chin C-S, Peluso P, Sedlazeck FJ, Nattestad M, Concepcion GT, Clum A, Dunn C, O’Malley R, Figueroa-Balderas R, Morales-Cruz A (2016). Phased diploid genome assembly with single-molecule real-time sequencing. Nat Methods.

[22] Quesneville H, Bergman CM, Andrieu O, Autard D, Nouaud D, Ashburner M, Anxolabehere D (2005). Combined evidence annotation of transposable elements in genome sequences. PLoS Comput Biol.

[23] Ragagnin GT, Bernardo LP, Loreto EL (2016). Unraveling the evolutionary scenario of the hobo element in populations of Drosophila melanogaster and D. simulans in South America using the TPE repeats as markers. Genet Mol Biol.

